# Alteration of the translational readthrough isoform AQP4ex induces redistribution and downregulation of AQP4 in human glioblastoma

**DOI:** 10.1007/s00018-021-04123-y

**Published:** 2022-02-20

**Authors:** Onofrio Valente, Raffaella Messina, Giuseppe Ingravallo, Emilio Bellitti, Domenico Sergio Zimatore, Luigi de Gennaro, Pasqua Abbrescia, Roberta Pati, Claudia Palazzo, Grazia Paola Nicchia, Maria Trojano, Francesco Signorelli, Antonio Frigeri

**Affiliations:** 1 Laboratory of Neurochemistry, Department of Basic Medical Sciences, Neurosciences and Sense Organs, School of Medicine, University of Bari Aldo Moro, Piazza Giulio Cesare, 70100 Bari, Italy; 2 Department of Emergency and Organ Transplant, School of Medicine, University of Bari Aldo Moro, Bari, Italy; 3 Interventional and Diagnostic Neuroradiology Unit, Polyclinic Hospital of Bari, Bari, Italy; 4 Department of Bioscience, Biotechnology and Biopharmaceutics, University of Bari Aldo Moro, Bari, Italy; 5 Dominick P. Purpura Department of Neuroscience, Albert Einstein College of Medicine, 840 Kennedy Center, Bronx, NY USA; 6 Division of Neurosurgery, Department of Basic Medical Sciences, Neurosciences and Sense Organs, School of Medicine, University of Bari Aldo Moro, Piazza Giulio Cesare, 70100 Bari, Italy

**Keywords:** AQP4 isoforms, Vasogenic edema, Orthogonal arrays of particles, Edema index

## Abstract

Glioblastoma multiforme (GBM) is characterized by a remarkable cellular and molecular heterogeneity that make the behavior of this tumor highly variable and resistant to therapy. In addition, the most serious clinical complication of GBM and other brain tumors is the development of vasogenic edema which dramatically increase the intracranial pressure. In the present study we evaluate the expression, supramolecular organization and spatial distribution of AQP4 and AQP4ex, the new readthrough isoform of AQP4, in relationship with the degree of vasogenic brain edema and tumor progression. To this purpose, tissue samples from regions of tumor core, peritumoral and non-infiltrated tissues of each GBM patient (*n* = 31) were analyzed. Immunofluorescence experiments revealed that the expression of AQP4ex was almost absent in tumoral regions while the canonical AQP4 isoforms appear mostly delocalized. In peritumoral tissues, AQP4 expression was found altered in those perivascular astrocyte processes where AQP4ex appeared reduced and partially delocalized. Protein expression levels measured by immunoblot showed that global AQP4 was reduced mainly in the tumor core. Notably, the relative amount of AQP4ex was more severely reduced starting from the peritumoral region. BN-PAGE experiments showed that the supramolecular organization of AQP4 is only partially affected in GBM. Edema assessment by magnetic resonance imaging revealed that the level of AQP4ex downregulation correlated with edema severity. Finally, the degree of BBB alteration, measured with sodium fluorescein content in GBM biopsies, correlated with the edema index and AQP4ex downregulation. Altogether these data suggest that the AQP4ex isoform is critical in the triggering event of progressive downregulation and mislocalization of AQP4 in GBM, which may affect the integrity of the BBB and contributes to accumulation of edema in the peritumoral tissue. Thus, AQP4ex could be considered as a potential early biomarker of GBM progression.

**Supplementary Information:**

The online version contains supplementary material available at 10.1007/s00018-021-04123-y.

## Introduction

Glioblastoma multiforme (GBM) is the most common primary malignant brain tumor in adults and although intense research has been conducted over the last 25 years, only modest advances in treatment have occurred. This is probably due to the fact that GBM is characterized by a remarkable cellular and molecular heterogeneity not only across but also within glioblastomas [4] Despite the intense research conducted to understand the molecular events occurring in GBM, most studies over the years have focused on the GBM core tumor area, whereas less is known about the peritumoral area, which is often infiltrated by cancer cells. Only recently have studies focused on the characterization of this area surrounding GBM that is not enhanced on magnetic resonance imaging (MRI) and is apparently “normal” under the microscope white light. Information on this region would permit better definition of its role in GBM progression and help search for more specific therapeutic targets.

One frequent and clinically significant complication of GBM is the development of edema, which dramatically increases intracranial pressure. Classically, GBM-associated cerebral edema is vasogenic in nature and it is characterized by failure of blood–brain barrier (BBB) integrity, resulting in accumulation of fluid in brain parenchyma and alteration of the cerebral microenvironment. Fluid accumulation is due to alteration of tight junction (TJ) proteins of the vascular endothelium and formation of new vessels that are disorganized, tortuous and extremely leaky. GBM also causes loss of astrocyte polarization, but whether and how loss of polarity is related to disturbance of microvascular TJ is unknown.

Water movement between compartments is determined by osmotic and hydrostatic pressure gradients and under the control of aquaporin water channels. The most important aquaporin expressed in brain parenchyma is aquaporin-4 (AQP4), which is highly expressed in astrocyte perivascular processes close to the BBB. Due to the great abundance of AQP4 and since astrocyte processes cover about 99% of the cerebral vasculature surface, it is conceivable that AQP4 could be involved in edema formation occurring in GBM.

Previous studies report conflicting results in which AQP4 levels increased [29] or decreased [26] in GBM. Furthermore, most GBM studies were not performed on fresh preparation but using fixed and paraffin included specimens, often limiting the analysis to immunohistochemistry [37, 45, 48] and rarely enclosed the internal correct control from less or unaffected regions from the same biopsy to determine the basal level of AQP4. Thus, since the expression levels of AQP4 change considerably between CNS regions [15] and within the same region between gray and white matter [41] the importance of performing the analysis in a contiguous region from the same biopsy is fundamental for an accurate evaluation.

AQP4 is expressed as a particular morphological feature called orthogonal array of particles (OAPs) visible by FFEM. These structures are aggregates of the tetrameric unit. AQP4 is expressed in different isoforms: two canonical M23 and M1 and two extended M23ex and M1ex, which influence expression, function and assembly in OAPs [5, 17, 34]. The extended isoforms are generated by the translational readthrough mechanism and are expressed in human, mouse and rat central nervous system (CNS). AQP4ex contains a C‐terminal extension of 29 amino acids from the canonical stop codon to a downstream more efficient stop codon [20]. AQP4ex is mainly confined to the perivascular astrocyte processes. Furthermore, phosphorylation of serine residues (Ser331 and Ser335) of AQP4ex seems to play an important role in the short-term regulation of channel gating and water permeability [6].

To evaluate the functional role of AQP4ex we recently generated a transgenic mouse model in which AQP4ex was completely abolished using the CRISPR-Cas9 technique [30]. AQP4ex-KO mice revealed that AQP4ex is indispensable to anchor AQP4 at the perivascular astrocytic endfoot membrane domains. Indeed, large OAPs made of M1 and M23 canonical isoforms, still abundantly expressed in the AQP4ex mouse, are delocalized and confined at the astrocytic processes facing the brain neuropile. More interestingly, AQP4ex is also necessary to generate the NMO-IgG epitope in mouse [30].

These data led us to suppose that AQP4ex may be involved in the alteration observed in the GBM. Thus, the purposes of this study are: (1) to determine differences in protein expression levels of AQP4 in brain parenchyma invaded by or surrounding the GBM core; (2) to determine if AQP4ex is affected and to what extent it is involved in AQP4 polarization and expression in GBM and (3) to correlate the level of alteration with the amount of vasogenic edema in GBM.

## Materials and methods

### Tissue collection and histological characterization

This study was approved by the local institutional review board (project. n 6898) and conducted in accordance with the Principles of Ethics for Medical Research Involving Human Subjects set out in the Declaration of Helsinki and its subsequent amendments. Demographic, clinical and histopathological data were prospectively collected and entered in the institutional database of brain gliomas. The salient features of the patient cohort used for this study are reported in Table 1.

**Table 1:** Features of the GBM patient cohort

Patient	Age	Gender	Brain region	Tumor (cm^3^)	Edema (cm^3^)	EI	EI
1	60	M	Fronto-parietal	11.89	62.77	6.279226241	> 2
2	73	M	Temporal	40.6	33.3	1.820197044	1–2
3	67	M	Posterior parietal	67.37	37.57	1.557666617	1–2
4	59	M	Frontal	53.08	70.09	2.320459683	> 2
5	78	F	Temporo-insular	22.63	81.98	4.622624834	> 2
6	45	M	Parieto-insulo-occipital	171.07	0	1	1
7	68	M	Fronto-insular	10.11	164.43	17.26409496	> 2
8	61	M	Temporo-parietal	104.95	60.49	1.5763697	1–2
9	61	M	Frontobasal parasagittal	35.17	65.25	2.855274382	> 2
10	55	F	Fronto-temporo-insular	67	14	1.208955224	1–2
11	53	M	Temporo-frontobasal-frontal	108.02	35.88	1.332160711	1–2
12	67	M	Temporo-insular-frontobasal	152.42	70.53	1.462734549	1–2
13	71	F	Temporo-parietal	45.65	2.36	1.0516977	1–2
14	53	M	Frontal	71.35	92.48	2.29614576	> 2
15	69	M	Temporo-occipital	96.98	36.96	1.381109507	1–2
16	63	M	Fronto-temporal	52.78	83.32	2.578628268	> 2
17	54	M	Posterior parietal	23.61	0.39	1.016518424	1–2
18	51	F	Temporo-parietal	2.89	0.31	1.107266436	1–2
19	64	F	Frontal	16.06	0	1	1
20	75	M	Frontal	40.74	126.06	4.094256259	> 2
21	72	M	Temporo-parietal	17.85	1.44	1.080672269	1–2
22	53	M	Temporal	7.8	84.13	11.78589744	> 2
23	54	F	Temporal	9.38	44.4	5.73347548	> 2
24	67	M	Frontobasal	4.45	8.64	2.941573034	> 2
25	56	F	Fronto-parietal	49.33	1.38	1.027974863	1–2
26	67	M	Temporal	55.55	0	1	1
27	61	F	Temporo-parietal	62.05	66.92	2.078485093	> 2
28	63	F	Temporal	37.06	34.12	1.920669185	1–2
29	6	F	Temporal	22.9	31.31	2.367248908	> 2
30	64	M	Temporal	67.01	105.14	2.569019549	> 2
31	60	M	Temporal	67.24	79.17	2.177424152	> 2

Tissues samples were obtained intraoperatively from regions of tumoral core (T), defined as zones clearly tumoral in white light under the microscope and corresponding to T1-weighted contrast-enhancing region on MRI. We also collected peritumoral (Pt), and non-infiltrated tissue (N), defined as zones of brain parenchyma surrounding T, apparently normal under the microscope in white light and in the absence of contrast enhancement (T_1_) in three-dimensional MRI, but respectively contiguous to T and showing hyperintense signal in T2-weighted MRI and in fluid-attenuated inversion recovery (FLAIR) [18] and non-contiguous to T, out of T2-weighted hyperintensity. We advocate supratotal glioma resection of non-functional brain parenchyma surrounding tumoral areas, in an effort to maximize tumor resection and increase patient survival [3, 32]. Each tissue sample was examined histopathologically to confirm gross tumor infiltration, peritumoral infiltration or absence of tumoral infiltration.

Tissues were fresh frozen in isopentane cooled in liquid nitrogen for 20 min after tumor resection and then stored in liquid nitrogen. Cryosections of 8 μm thickness were cut on a cryostat (CM 1900; Leica, Wetzlar, Germany) at − 20 °C and immediately used to perform hematoxylin–eosin (H&E) staining, in order to evaluate the histological features of each tissue. By using Papanicolaou Harris’ hematoxylin solution (Carlo Erba, Italy), nuclei were stained in blue, and after washing, eosin (Carlo Erba, Italy) solution was used to stain cytoplasmic proteins in pink, and finally washed. Then, cryosections were de-hydrated in graded ethanol, fixed with xylene solution (PanReach Applichem, Darmstadt, Germany), and mounted with Canada Balsam (Millipore, Burlington, Massachusetts, USA). Sections were then observed under a LEICA EL6000 microscope, with a 20×/0.55 HC PL FLUOTAR objective.

### Magnetic resonance imaging (MRI) and edema index calculation

All images were acquired on a 1.5 Tesla MRI scanner using a standard coil. The volumes of high-grade gliomas and the volumes of surrounding edema were quantified on MRI imaging by an expert neuroradiologist (DSZ with 13 years of experience).

The image datasets used consisted of (1) T1-weighted, contrast-enhanced (gadolinium) (T1c) images with 3D acquisition and isotropic voxel sizes of 1 mm for all patients. (2) FLAIR images with 3D acquisition and isotropic voxel sizes of 1 mm for most patients or, alternatively, T2-weighted images with 2D axial acquisitions and thickness of 5 mm. T1c were used for tumor segmentation, while FLAIR/T2 sequences (highlighting differences in tissue water content) were chosen to better define edema and, if present, a tumor component lacking enhancement.

Tumor and edema volumes were calculated using three-dimensional (3D) Slicer Software (Release 4.10.2). T1c images were spatially aligned and co-registered to FLAIR/T2 weighted images. Then an interactive segmentation algorithm was adopted. Enhanced solid portions of tumors and necrotic nuclei were initially delineated "slice by slice" on T1c images using the “3D Slicer Level Tracing” tool; in a second phase manual corrections were made with the “Paint” and “Erase” tools. The edema was manually delineated "slice by slice" with the “Paint” and “Erase” tools; the same method was used to segment the tumor core without enhancement when it was present. At the end of the process, the solid portions with and without enhancement and the necrotic nucleus of the tumor were considered together as "overall tumor nucleus" as opposed to "edema".

To quantitatively evaluate the degree of brain edema in GBM patients the edema index parameter (EI) was calculated as tumor volume + edema volume/tumor volume [16, 26].

### Antibodies

For the immunoblot and for immunofluorescence experiments the following primary antibodies were used: rabbit anti-human AQP4ex [6] used at a concentration of 0.3 μg/mL, custom rabbit anti-AQP4 were generated using the c-terminus sequence as previously described [10] (GenScript Biotech, Piscataway, NJ, USA) used at a concentration of 0.13 μg/mL for immunoblot and at 0.4 μg/mL for immunofluorescence. The secondary antibody used for immunoblotting experiments was anti-rabbit IgG-HRP (Bio-Rad Cat# 172–1019, RRID:AB_11125143) and for immunofluorescence AlexaFluor 488 anti-rabbit was used at a concentration of 1 μg/mL (Life Technologies, Thermo Fisher Scientific Cat# A-11034, RRID:AB_2576217).

### Sample preparation for SDS-PAGE and BN-PAGE

Tissues were dissolved in seven volumes of BN buffer (1% Triton X-100, 12 mM NaCl, 500 mM 6-aminohexanoic acid, 20 mM Bis–Tris, pH 7.0, 2 mM EDTA, 10% glycerol) added with protease inhibitor cocktail EDTA-free (Roche, Basel, Switzerland). After tissue lysis on ice for 1 h, samples were centrifuged at 17,000×*g* for 30 min at 4 °C, and then the supernatants were collected to evaluate total protein content by using a BCA Protein Assay Kit (Thermo Scientific, Waltham, Massachusetts). BN-PAGE experiments were performed as previously described [24]. Briefly, 40 μg of protein sample was mixed with 2 μL of loading buffer (5% of Coomassie Blue G-250, 750 mM aminocaproic acid) and 10% glycerol by volume and then loaded onto polyacrylamide native gradient gel (3–9%). Anode buffer (25 mM imidazole pH 7.0) and blue cathode buffer (50 mM tricine, 7.5 mM imidazole, 0.02% Coomassie Blue G-250) were used as running buffers and the electrophoresis was performed at 6 mA at 4 °C. After the line of Coomassie Blue G-250 dye reached half of the gel, blue cathode buffer was substituted by cathode buffer (50 mM tricine, 7.5 mM imidazole). At the end of gel running, proteins were transferred to PVDF membranes (Millipore, Burlington, Massachusetts, USA) for immunoblot analysis, as described below.

As previously described [30], for SDS-PAGE experiments 20 μg of homogenates was dissolved in Laemmli sample buffer 2× (Bio-Rad, California, USA) added with 50 mM dithiothreitol (DTT) and, after denaturation, loaded onto a 13% polyacrylamide gel; TGS (Trizma, glycine and SDS) was used as running buffer. After electrophoresis, proteins on gel were transferred to polyvinylidene difluoride (PVDF) membranes (Millipore, Burlington, Massachusetts, USA) for immunoblotting. Transfer was verified with staining of the membrane with Rouge Ponceaux.

### Immunoblotting

PVDF membranes were incubated with primary antibodies prepared in 5% non-fat milk, washed, and incubated with peroxidase-conjugated secondary antibody. Reactive proteins were revealed using an enhanced chemiluminescent detection system (Clarity Western ECL Substrate, Bio-Rad, California, USA) and visualized on a Chemidoc Touch imaging system (Bio-Rad, California, USA). Densitometry analysis was performed using Image Lab (Bio-Rad California, USA), and the relative expression of proteins was normalized with Rouge Ponceaux staining. AQP4 and AQP4ex expression of PT and T tissues was represented as the percentage change from N control tissues, set on 100%. Moreover, the relative percentage of M1ex and M23ex was calculated in each tissue in order to study the ratio between extended isoforms. Finally, the percentage of M23ex relative to global AQP4 (M23ex + M1 + M23) was calculated in each tissue.

### Immunofluorescence

Immunofluorescence experiments were performed as previously described [30]. Briefly, 8 μm cryosections, collected on SuperFrost Plus adhesion slides (Thermo Fisher Scientific, Waltham, Massachusetts, USA), were re-hydrated in PBS for 10 min, fixed in 4% PFA solution for 10 min and then blocked with PBS-Gelatin 0.1% for 15 min at room temperature. Cryosections were incubated with primary antibodies in PBS-Gelatin 0.1% for 1 h at room temperature, washed for 15 min and then incubated with secondary antibodies prepared in the blocking solution in the dark for 1 h at room temperature. Finally, the sections were washed for 15 min in PBS and mounted with Mowiol (Sigma-Aldrich) added with DAPI (4′,6-diamidino-2-phenylindole, Life Technologies, Thermo Fisher Scientific, Waltham, Massachusetts, USA). The images were finally obtained under an SP8 confocal automated inverted Lightning microscope (Leica TCS) using 20×/0.55 HC PL FLUOTAR objectives or with a 63X HC PL Apo oil CS2 objective.

### Evaluation of sodium fluorescein concentration in GBM biopsies

Sodium fluorescein (SF) (Monico Spa, Italy) is a fluorescent dye, largely used for the guide-resection of high-grade gliomas [7, 31]. SF solution (200 mg/mL) was used to build a calibration curve useful for determining the concentration (pg/mL) of fluorophore in biopsies. Fluorescence was measured by an automatic microplate reader (FLEX Station, Molecular Devices) both in GBM samples SF-labeled and in non-fluorescent samples as negative controls. 40 μL of each sample was placed in 96-well black walls-clear bottom microplates (Corning, NY, USA) and the fluorescence was acquired by using SoftMax Pro software.

### Statistical analysis

All immunoblot data are reported as a violin plot with the median; data about SF concentration are represented as a scatter plot with the median. Statistical analysis was performed using GraphPad Prism (GraphPad, San Diego, CA, USA). Data distribution was analyzed using the Shapiro–Wilk test: on parametric data the analysis of variance (one-way ANOVA) followed by Tukey’s post-test was performed, while on non-parametric ones one-way ANOVA on ranks (Kruskal–Wallis test) followed by Dunn’s multiple comparisons test was performed. *p* value < 0.05 was considered statistically significant.

## Results

### AQP4 localization is affected in histologically characterized GBM samples

**Fig. 1:**
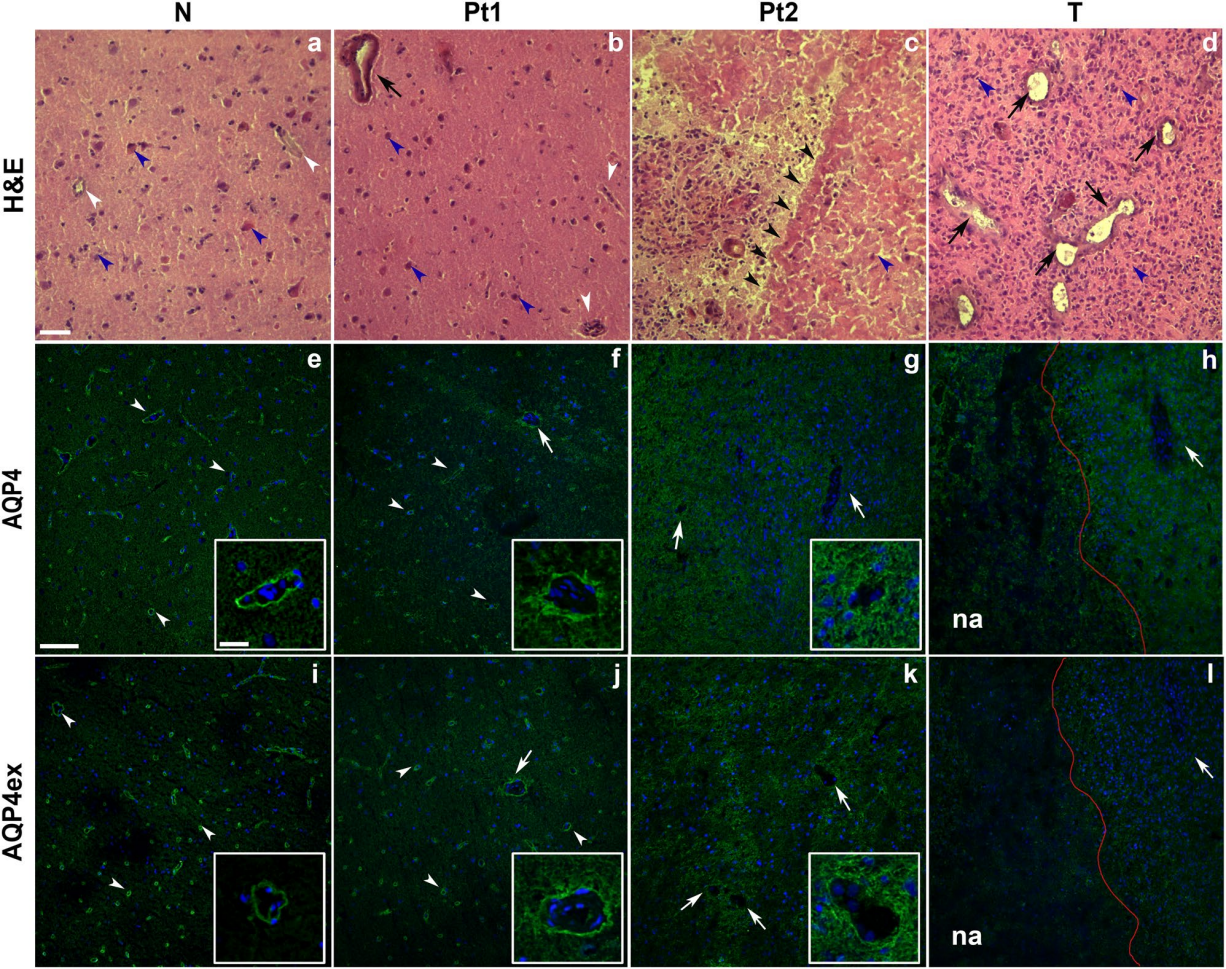
AQP4 localization in histologically defined GBM samples. **a**–**d** Hematoxylin–eosin (H&E) staining of non-infiltrated tissue (N) peritumoral (Pt 1–2) and tumoral tissues (T): **a** cryosection of typical non-infiltrated tissue displays low cellularity, normal nuclei (blue arrowhead) and normal vessels (white arrowhead),characterized by small lumen and regular endothelia. Pt (**b**, **c**) is characterized by different areas that underline the intermediate characteristics of this tissue; Pt1 (**b**) shows normal vessels (white arrowhead), low cellularity and normal nuclei (blue arrowhead) comparable to unaffected tissue. However, few vessels (black arrow) display altered shape, underling some particular features of GBM progression. In the Pt2 region (**c**) near the tumor note an area with low cellularity (blue arrowhead) in which the tumor migration front is observable. **e**–**l** Immunofluorescence localization of AQP4 and AQP4ex in non-infiltrated tissue (N) peritumoral (PT 1–2) and tumoral tissues. Note the perivascular staining of AQP4 and AQP4ex in N (arrowheads and insets), while the perivascular AQP4ex staining is absent in T (**l**, arrow) and AQP4 is redistributed on the whole parenchyma (**h**). Cell nuclei were stained with DAPI (in blue). The red line in T indicates the border between the necrotic area (na) and the non-necrotic area of the tumor region**.** High cellularity of tissue is confirmed by numerous nuclei stained with DAPI (blue). Pt shows perivascular staining of AQP4 and AQP4ex (arrowheads) in non-infiltrated areas (Pt1) with few capillaries showing a slight peripheral staining around vessels (arrows) more visible at higher magnification (inset). Nearly complete redistributed staining of AQP4 is observed in the infiltrated Pt2 area **(**see inset**)**. Scale bar 100 µm, inset 20 µm

Histopathological characterization of biopsies by hematoxylin–eosin staining showed extreme heterogeneity of GBM-infiltrated tissue, (Fig. 1). The tissue material deriving for each biopsy was then evaluated and classified in non-infiltrated (N), peritumoral or invasion front (Pt) additionally classified in peritumoral 1 (Pt 1) and peritumoral 2 (Pt 2) and tumor core (T) (Fig. 1, top). N showed vessels with normal endothelial pathway, low cellularity with small and regular cellular elements and absence of necrosis (Fig. 1a). T was characterized by high cellular density lacking an architectural design, with areas of necrosis, hemorrhagic foci and a high number of vessels, of irregular shape and larger size. These vessels did not show a linear aspect but appeared distributed in an uneven and messy way (Fig. 1d). Pt samples were characterized by neoplastic areas and nearby healthy brain parenchyma, thus presenting intermediate features between N and T. In particular, Pt1 was the region having similar histological characteristics to unaffected tissue (low cellularity). Blood vessels appeared generally normal with few slightly altered vessels in some areas of this region (Fig. 1b). Pt2 was characterized by a transitional zone, containing a migration front of neoplastic cells invading healthy brain parenchyma (Fig. 1c). These findings suggest that the peritumoral tissue shows signs of reorganization and transformation. Given the great heterogeneity of the preparations, the peritumoral tissue was defined as a tissue that had at least 40–50% of clearly distinguishable tumor tissue compared to a macroscopically non-infiltrated tissue.

It has been amply demonstrated that AQP4 is localized at the astrocyte perivascular end-foot membrane [11, 27]. Furthermore, recent data show a crucial role of AQP4ex for the correct localization of the canonical AQP4 isoforms at the BBB domain [30]. Literature also reports that AQP4 is affected in GBM condition [46]. To morphologically evaluate localization of AQP4 and AQP4ex in tissue samples, immunofluorescence experiments were performed (Fig. 1, middle-bottom). Expression of AQP4ex in T samples was almost absent in perivascular and non-perivascular astrocytes processes (Fig. 1l), while the canonical AQP4 isoforms appeared absent at the perivascular pole and mostly delocalized in the astrocyte processes away from brain microvasculature (Fig. 1h). Most interesting was the analysis of Pt samples (Fig. 1, PT1–2). In Pt1 region, brain capillaries showed some vessels with partially delocalized AQP4ex staining (Fig. 1j) together with a moderate and partially delocalized staining of AQP4 canonical isoforms (Fig. 1f). Staining of AQP4 was increased in the neuropile astrocyte processes. Furthermore, in transitional regions of Pt2, AQP4ex (Fig. 1k) and AQP4 (Fig. 1g) staining appeared to be disposed in a radial pattern surrounding the vessels indicating a marked and localized redistribution of AQP4.

### AQP4 protein expression is reduced in GBM biopsies

**Fig. 2:**
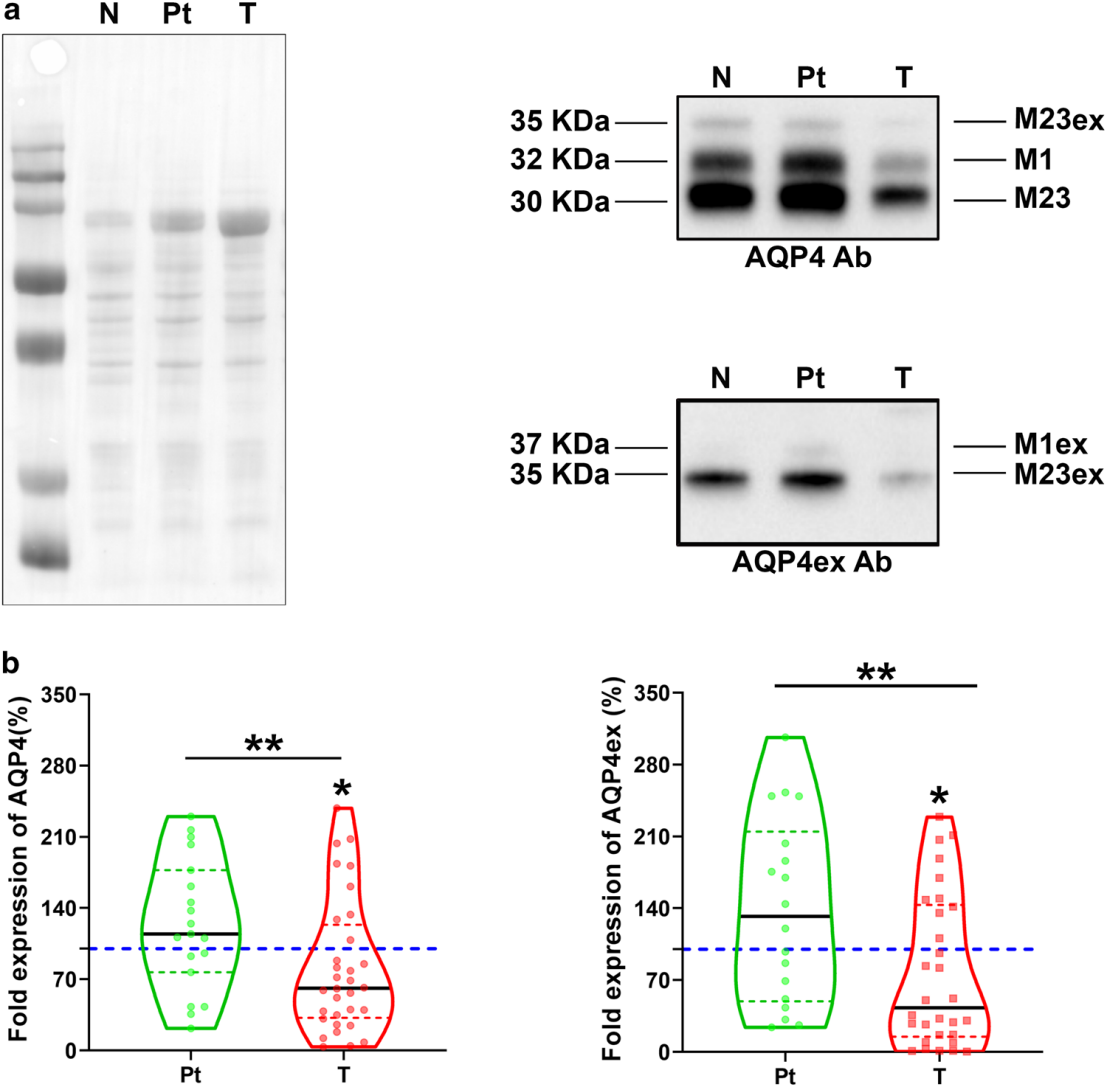
Immunoblot analysis of AQP4 and AQP4ex levels in GBM biopsies. **a** Left: Rouge Ponceaux staining of total proteins of N, Pt and T samples transferred on PVDF membranes after SDS-PAGE. Right: immunodetection of the four AQP4 isoforms of 30 kDa (M23), 32 kDa (M1), 35 kDa (M23ex) and 37 kDa (M1ex) revealed with global AQP4 (top) and AQP4ex specific antibodies (bottom); **b** violin plots showing data distribution and the continuous black line represents the median fold change in the expression of AQP4 (left) and AQP4ex (right) expression in Pt and T tissues, compared to N samples (dotted blue line) seats at 100%. Green and red dotted lines represent the upper and lower quartile in each distribution (*p < 0.05, Kruskal–Wallis–Dunn's multiple comparisons test)

To investigate protein expression levels of AQP4 according to degree of tumoral infiltration, immunoblot experiments were performed on histologically classified portions of each type of tissue samples. Immunoblot was performed using AQP4 global antibody and AQP4ex specific antibody (Fig. 2). Densitometry analysis revealed that the overall quantity of AQP4 was not substantially modified in Pt compared to N regions, although a moderate increase was observed. On the contrary, the total amount of AQP4 was slightly but significantly reduced in T areas compared to Pt and N regions. Notably, the relative amount of AQP4ex compared to total AQP4 levels was more severely reduced in T samples. This suggests that the AQP4ex isoform could be an initial factor that determines overall AQP4 reduction.

**Fig. 3:**
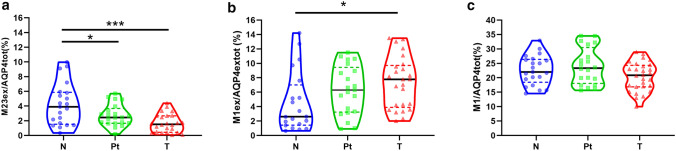
AQP4 isoform content in GBM samples control (N), peritumoral (Pt) and tumoral (T) regions. **a** Violin plots reporting data distribution of the M23ex isoform amount relative to the total AQP4 content measured by immunoblotting. **b** Plots reporting M1ex isoform (37 KDa) content relative to the total AQP4ex measured by immunoblotting using anti-AQP4ex antibody. **c** M1 expression levels. Blue, green and red dotted lines represent the upper and lower quartile in each distribution. **a** ANOVA-one way, Tukey's multiple comparisons test, *p < 0.05, ***p < 0.0001. **b**, **c** Kruskal–Wallis, post-test: Dunn's multiple comparisons test, *p < 0.05

To elucidate this aspect, AQP4ex levels were evaluated in association with the total levels of AQP4. AQP4ex protein levels were highly variable in N samples of different biopsies (range 2–10%), probably in relation to the highly regulated role of this isoform in water transport [6]. Importantly, the amount of AQP4ex compared to the total amount of AQP4 were reduced significantly already Pt regions and became very low in T regions (see Fig. 3). Densitometry analysis of the two AQP4ex isoforms (M23ex and M1ex) in N regions showed a ratio of 19:1 very different to that found (3:1) for the canonical isoforms (M23 and M1), indicating a different translation mechanism of mRNA for the two extended isoforms. Interestingly, reduced expression of AQP4ex (mainly due to M23ex) is coupled to an increase of the M1ex isoform and consequently the M23ex/M1ex ratio is reduced to approximately 13:1. Conversely, no changes were observed in the canonical isoform ratio as the M1 content emerged unaltered. These data suggest that the increase of the M1ex content may contribute to the instability of AQP4ex at the perivascular pole and to its downregulation.

### Edema index underlines the role of AQP4ex in GBM

**Fig. 4:**
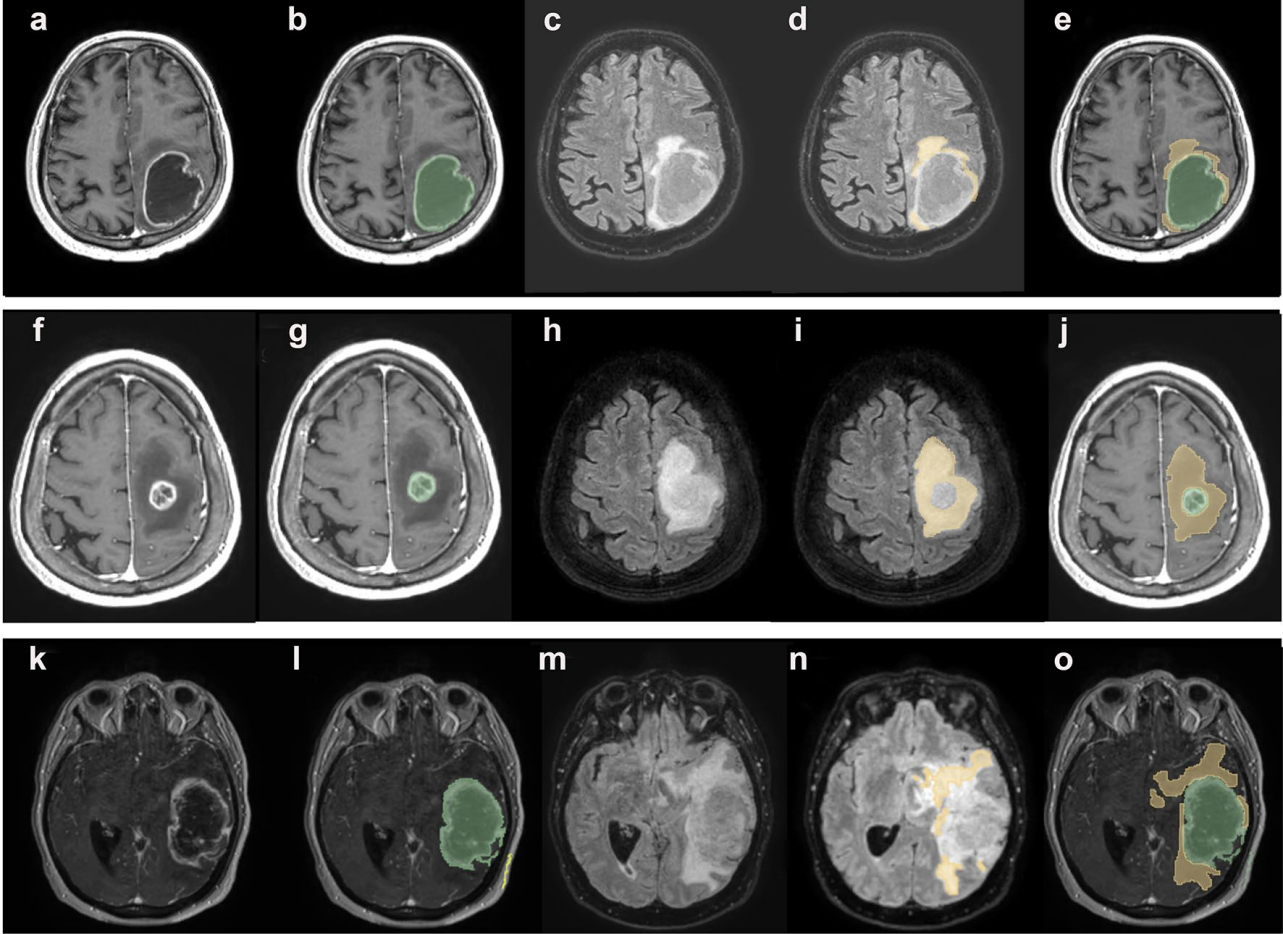
MRI of GBM biopsies for each edema index (EI) category. In the first row a glioma with EI approximately = 1; in the second row a glioma with EI > 2; in the third row a glioma with 1 < EI < 2. In **a**, **f**, **k** T1 with gadolinium sequence. In **b**, **g** and **l** tumor volume was segmented in green. In **c**, **h** and **m** FLAIR sequences, co-registered with T1 with gadolinium. In **d**, **i** and **n** edema volume was segmented in yellow. In **e**, **j** and **o** tumor and edema volume segmentation is represented on a T1 with gadolinium acquisition

**Fig. 5:**
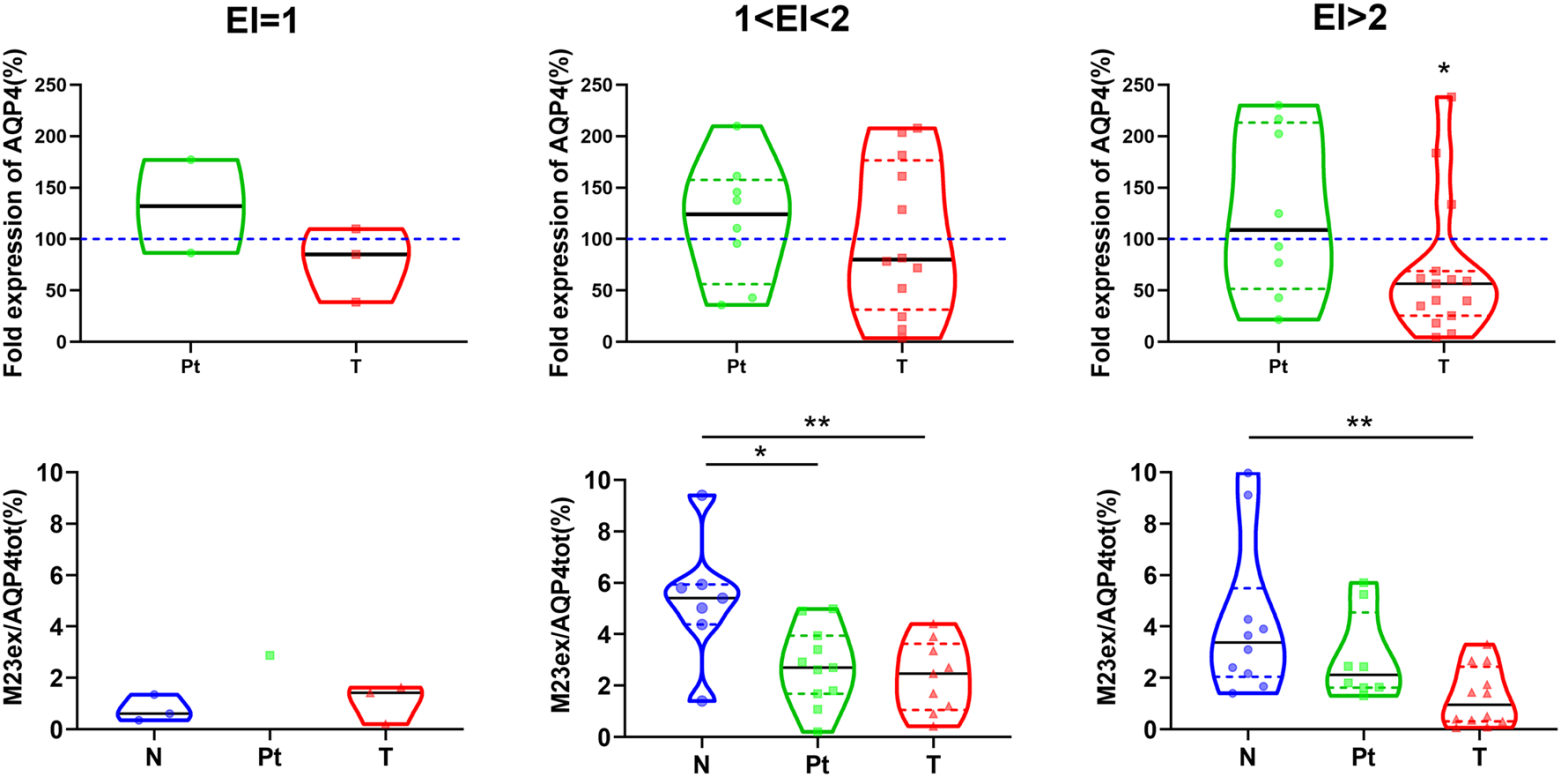
Expression of AQP4 and AQP4ex in correlation with the edema index (EI). Top: violin plots reporting AQP4 expression levels in the GBM regions (Pt and T) of each EI category compared to N samples (dotted blue line) seats at 100%. Bottom: relative amount of the M23ex isoform to the global AQP4 expression in the same GBM samples. Blue, green and red dotted lines represent the upper and lower quartile in each distribution. *p < 0.05, **p < 0.001: Kruskal–Wallis, post test: Dunn's multiple comparisons test

Expression levels of AQP4 were correlated with the edema index (EI), a parameter previously used to evaluate the extent of edema correlated to tumor volume [16, 23, 26]. Three different EI categories were established based on the evaluation (see methods) of MRI images (Fig. 4): EI = 1 (no edema); EI = 1 < EI < 2 (moderate edema) EI > 2 (severe edema). The large majority (about 90%) of our GBM cases showed moderate or severe edema (Fig. 5), with only few cases having no edema (EI = 1). The proportion between the two edema categories was approximately the same.

These data confirm the predominance of peritumoral brain edema in GBM and suggest an involvement of AQP4 in the brain edema occurring in GBM patients.

To evaluate the possible contribution of altered expression levels of AQP4 in peritumoral edema formation, protein levels were assessed in association with EI categories. Immunoblot analysis (Fig. 5) revealed a strong reduction of AQP4ex in both the moderate and severe edema categories, while the level of total AQP4 were reduced only in EI > 2. Notwithstanding the limited number of GBMs associated with the EI = 1 category, expression of total AQP4 and AQP4ex appeared not to be substantially affected compared to the control. Furthermore, differently to AQP4, protein content of AQP4ex appeared strongly reduced in Pt region of GBMs with moderate edema and a similar trend (although non-statistically significant) was observed in GBM with severe edema. Since, AQP4 was not altered in both Pt and T regions of GBMs with moderate edema, while AQP4ex was downregulated in both regions, we can conclude that AQP4ex is primarily involved in edema formation in GBM. This finding is also supported by a strong AQP4ex reduction observed in GBMs with severe edema.

Downregulation was not found to be correlated with tumor location (see supplementary Fig. 1).

### Supramolecular organization of AQP4 is partially affected in GBM

**Fig. 6:**
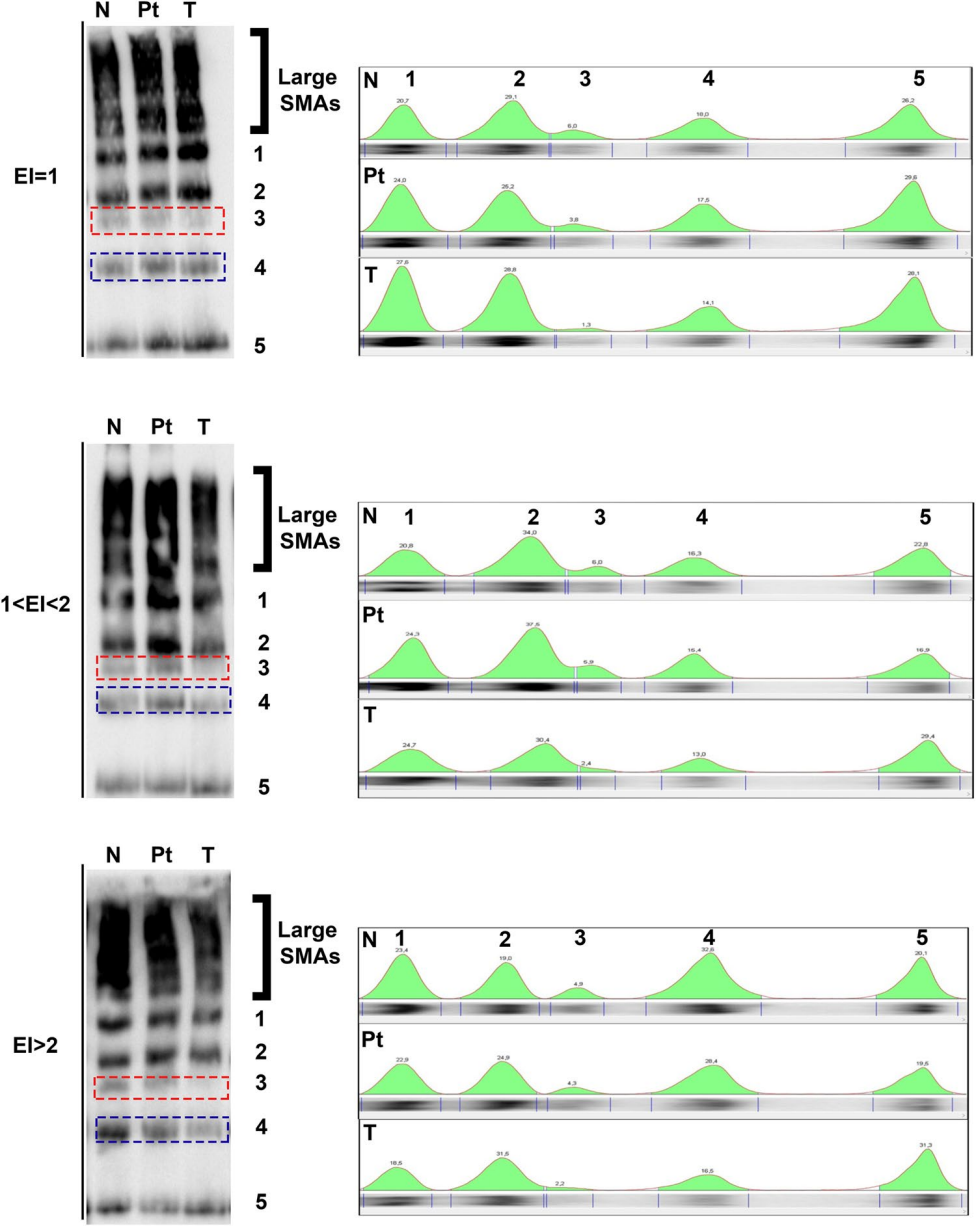
AQP4 supramolecular organization in GBM biopsies. Left: immunoblot after BN-PAGE of control (N), peritumoral (PT) and tumoral (T) tissues is shown for a representative case of each EI category using AQP4 global antibodies. In addition to very high molecular size AQP4 large supramolecular assemblies (large SMAs), five different AQP4 pools (1–5) are indicated and quantified with the analysis of lane profile (right*).* Red and blue boxes highlight the affected pools (3–4) in T compared to the N, particularly in the edema containing EI categories

It is widely documented that supramolecular organization of AQP4 in OAPs is central for the correct localization of AQP4 at the perivascular pole. To evaluate AQP4 supramolecular organization in OAPs we performed BN-PAGE experiments, a biochemical technique largely used to evaluate the plasma membrane composition and number of the different AQP4 pools [24, 36]. Figure 6 shows the expression levels and aggregation state of AQP4, evaluated by immunoblot, of a representative GBM for each EI group using the three different tumor regions. Moderate changes in AQP4 pools abundance were observed with tumor progression. In particular, two AQP4 pools (red box for pool number 3, blue box for pool 4) were often slightly affected in GMB biopsies as their abundance reduced progressively starting in peritumoral region. This specific reduction of AQP4 pools, occurring in the edema associated GBM, indicates a selective alteration of AQP4 plasma membrane supramolecular organization. Interestingly, reduction in specific AQP4 pools correlated with the increase in EI values.

These results in line with the findings of SDS-PAGE experiments shown above, indicate that the observed alteration in AQP4 pool composition, as a consequence of AQP4ex strong downregulation, determine down regulation of AQP4 and delocalization of AQP4 from the perivascular pole to the brain parenchyma occurring during tumor development.

### BBB integrity is strongly altered in GMB

**Fig. 7:**
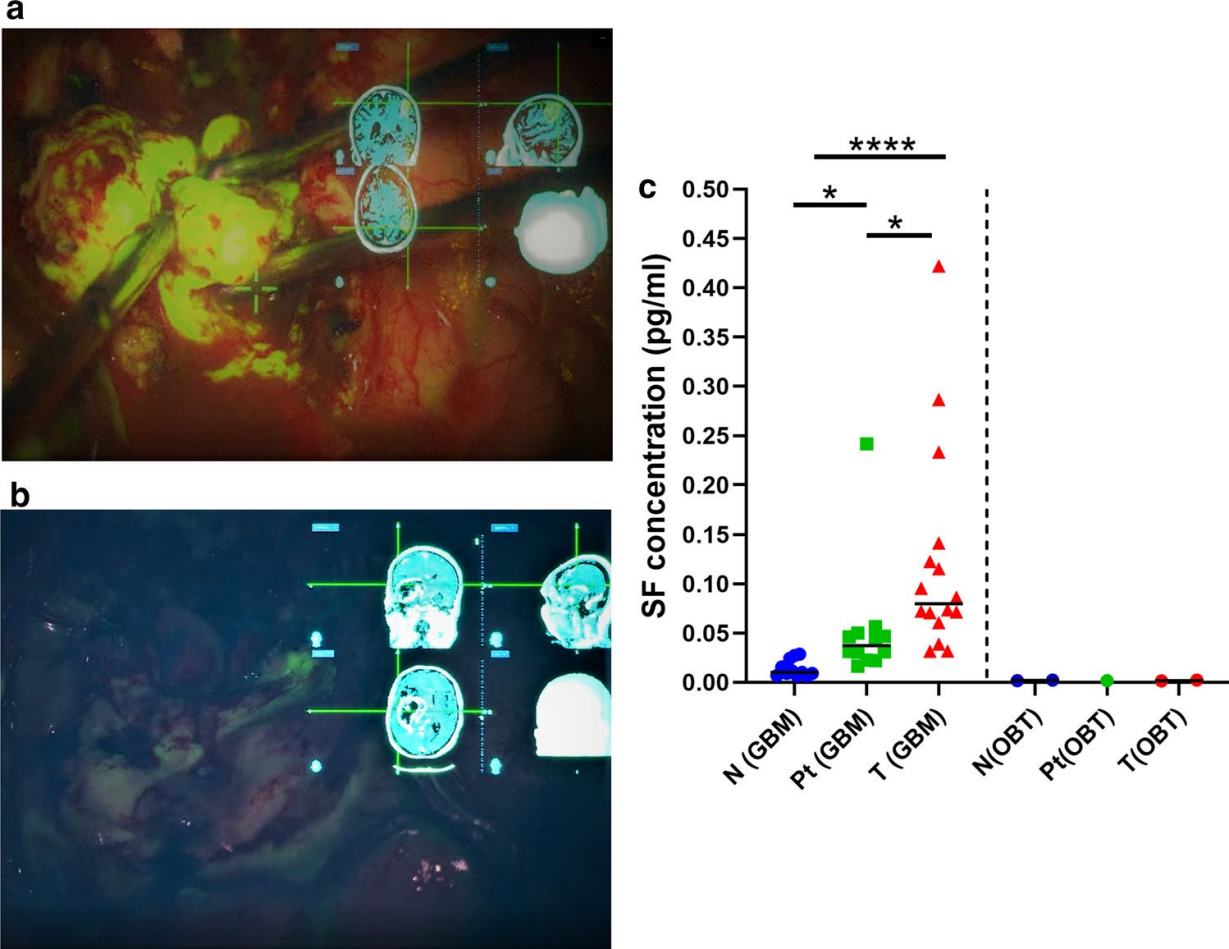
Use of SF in GBM resection and BBB integrity evaluation. **a** Intraoperative video frame picture during the surgical removal of the tumor (GMB grade IV WHO) after injection of SF and after the resection (**b**). Note the intense fluorescence in **a** and a residual fluorescence in the peritumoral tissue. On top right a projection of MRI obtained using intraoperative neuronavigation 3D reconstruction. **c** SF concentration measured in N, Pt and T samples of GBM and two low grade tumor tissues (OBT), namely astrocytoma and ependymoma (right to vertical dotted line). ***p < 0.0001; Kruskal–Wallis; Dunn’s multiple comparisons test

Sodium fluorescein (SF) is a fluorescent dye largely employed to evaluate intraoperatively the spread of tumor cells guiding surgical resection of high-grade gliomas [1, 7, 21] (see Fig. 7a, b). To investigate the extent of BBB alteration, we assessed the amount of SF in T, Pt and N regions of GBMs and also compared to non-treated samples (Fig. 7). Although a consistent variability of dye accumulation was found among T region samples, indicative of heterogeneity of tissue accumulation of SF in GBMs, SF content analysis showed a significant difference between tumor-infiltrated and unaffected tissue, indicating a leakage of BBB in T regions, resulting in dye extravasation and accumulation in brain parenchyma. Importantly SF levels in Pt regions also showed a statistically significant increase compared to N regions indicating a BBB alteration already occurring at this level. Additionally, in one diffuse astrocytoma and in one ependymoma SF intensity was very low suggesting no alteration of BBB. Of note, SF levels in unaffected regions were similar to SF levels in fluorescein-free N regions samples, indicating no extravasation of the dye in unaffected tissue or non-specific binding of the dye to brain parenchyma confirming the usefulness of SF in surgical resection (see supplementary Fig. 2).

## Discussion

One of the characteristics of GBM is its great invasiveness of the surrounding nervous tissue. However, studies conducted at the cellular and molecular levels suggest that GBM displays a pronounced biological and genetic heterogeneity between different tumors and within the same tumor, which makes the behavior of this tumor highly variable and resistant to therapy [43]. Thus, to identify factors involved in tumor aggressiveness and progression, it could be informative to consider variation of molecular expression according to different density in tumoral cells. In the present study we evaluated the difference of expression and spatial distribution of AQP4 in grossly tumoral, peritumoral or non-tumoral brain regions. In particular, the analysis of peritumoral regions proved to be highly informative for the present study.

### AQP4 expression is down regulated in tumoral tissue samples

Our data demonstrate that the levels of total AQP4 are substantially reduced in the tumoral core compared to Pt regions. The most striking difference was observed for AQP4ex protein levels, which were drastically reduced in T regions. Since AQP4ex is necessary to anchor AQP4 at the perivascular pole, as demonstrated in AQP4ex-KO mice, and having observed a correlation between the levels of AQP4ex alteration and the degree of AQP4 delocalization and reduction we could conclude that the strong reduction in AQP4ex causes the redistribution of AQP4 found in GBM.

At least two factors could be involved in AQP4 alteration in GBM: the tumor microenvironment and the extracellular matrix. Decreased expression of AQP4 in T regions could be related to the particular tumor microenvironment [9] where low tumoral tissue oxygenation (hypoxia) promotes the spread of cancer cell spreading into healthy brain tissue in order to escape the hostile environment. Hypoxia condition is due to an increased tumor cells proliferation that, together with tumor neoangiogenesis, characterized by proliferation of vessels lacking pericyte coverage and containing thicker basement membrane, determine inadequate intratumoral oxygenation. Several studies have reported that acute and chronic hypoxia influence AQP4 protein expression in both pathological [19] and physiological situations [8]. One of the major regulators of the adaptive response of hypoxia are the HIF transcription factors, which regulate a multitude of genes involved in many different physiological processes [38]. Interestingly, previous studies showed that AQP4 could be a hypoxia responsive gene through HIF-1alpha [33]. In particular, cultured astrocytes exposed to hypoxia conditions gradually decrease AQP4 protein expression [47]. Thus, hypoxia may be considered a triggering factor for AQP4 downregulation in astrocyte endfeet in GBM. It remains to be elucidated through which mechanism AQP4 is down regulated in hypoxia and how and to what extent AQP4ex is involved.

The extracellular matrix and in particular its component agrin, has been shown to be an important factor for AQP4 perivascular localization [46]. Since degradation and remodeling of the extracellular matrix are essential processes for cancer cell invasion and involve degradation of agrin, it is conceivable that AQP4 localization and expression is consequently affected. However, the absence of agrin could contribute but is unlikely to be the principal cause of AQP4 alteration since the hypoxic effect in astrocyte primary culture could be found without the contribution of agrin.

Finally, other astrocyte proteins could be involved in AQP4 protein downregulation such as those of the dystrophin glycoprotein complex which have been shown to be necessary for AQP4 expression at the perivascular pole [2, 12, 13, 25]. In particular dystrophin (DP71 isoform) as well as α-syntrophin, which are both required to anchor AQP4 at the vascular pole, may also be affected in GBM.

### AQP4 aggregation state is slightly altered in GBM

AQP4 is expressed in perivascular astrocyte processes in extremely large OAPs. Previous freeze-fracture studies report that the OAP density in GBM is very low [29], similar to that found in mouse or rat brain parenchyma away from the perivascular astrocyte endfoot [35] indicative of the fact that AQP4 could be dissociated from the OAPs and redistributed across the entire surface of the cells. Our BN-PAGE data show that the supramolecular organization of AQP4 is only partially affected in GBM. Indeed, although the number of AQP4 pools (i.e., OAPs) is mainly retained in T regions, the fine composition of some AQP4 supramolecular arrangements is altered. Mainly two AQP4 pools appeared to be quantitatively reduced compared to N regions. A similar situation occurs in AQP4ex-KO mice, in which the absence of AQP4ex alters AQP4 pool size and results in AQP4 delocalization [30]. This suggests that alteration of AQP4ex in GBM modifies the fine composition of AQP4 pools which affect both expression and polarization of AQP4. Thus, although limited to the AQP4 pools detected with the BN-PAGE, we can conclude that the capacity of AQP4 to aggregate is not grossly impacted and is not related to tumor progression. This contrasts with previously published findings using FFEM to evaluate OAPs distribution in GBM [46]. However, it should be considered that BN-PAGE data are not easily comparable with those from electron microscopy. Considering that the biochemical technique allows large tissue regions to be analyzed, which in turn allows a more complete view of the tissue under examination, whereas FFEM only allows very limited areas to be analyzed in detail. Thus, it is possible that regional differences in AQP4 aggregation could have been missed with the BN-PAGE analysis.

Quantitative analysis of AQP4ex in N regions of GBM samples indicate that AQP4ex protein expression levels in human brain are highly variable (between 2 and 10% of the total AQP4). Although, AQP4ex expression is low, its alteration determines a strong downregulation in the total amount of AQP4, indicating that AQP4ex is interacting with the canonical isoforms to spatially confine large amount of AQP4 at the perivascular astrocyte processes, in agreement with data obtained from studies performed in mice and rats [30].

Our immunofluorescence data show increased AQP4 staining of GBM infiltrated brain parenchyma, in which glioma cells reside. Considering that AQP4 expression is low or absent in glioma primary cell cultures [22, 28, 42], residual staining of AQP4 observed in the tumor core is most likely not associated to glioma cells but instead to the delocalization of AQP4 in astrocyte processes not coupled to blood vessels. Although a more detailed analysis is necessary, our results suggest that glioma cells in tumor tissue are likely not to express high levels of AQP4. In support of this conclusion, it should be considered that AQP4-OAPs are not compatible with migration of glioma cells and their survival [39]. However, it should be considered that studies on AQP4 migration and polarization were performed using cells transfected with the canonical isoforms [40], which should be revisited in the light of the role of AQP4ex in AQP4 aggregation and polarization in vivo.

### AQP4 downregulation in GBM progression is associated with vasogenic edema

It has been reported that BBB integrity is affected in GBM and this causes vasogenic edema [46]. Indeed, GBM is often associated with peritumoral brain edema, which results in increased intracranial pressure that may cause ischemia, herniation and eventually death. Based on capability of fluorescein to cross the leaky BBB in GBM this fluorescent molecule is often used for intraoperative identification of infiltrated tissue. Our results show that the T regions of the GBMs display an extremely high concentration of fluorescein, indicative of a robust dye extravasation and thus of intense BBB damage. Interestingly, fluorescein levels in Pt regions were also significantly increased compared to N regions but less compared to T regions, indicating the presence of peritumoral edema. All these data suggest that the major contributions in edema formation derive from the leaky vessels located in T and Pt regions and that fluid accumulates in these regions and propagates in the surrounding brain parenchyma.

Our data further support the conclusion that AQP4 alteration in GBMs may be an additional factor contributing to edema formation. This is confirmed by several of our findings: (1) the level of AQP4 alteration correlates with the EI, since AQP4 and AQP4ex are both highly downregulated in EI > 2; (2) AQP4ex is also downregulated in Pt regions with 1 > EI > 2; and (3) where no edema is present (EI = 1) AQP4 appears not to be altered. The reduction of AQP4ex in Pt regions with moderate edema suggests that AQP4ex could be the triggering event of progressive downregulation of AQP4 in the GBMs. Indeed, since AQP4ex is necessary to anchor AQP4 at the perivascular pole, its alteration could destabilize AQP4 at the BBB level and initiate the degradation process of mixed complexes of AQP4ex–AQP4 that normally reside at the BBB level. Hence, tumor development generates a process that also affects AQP4ex expression at the BBB. For instance, the interaction with the cytoskeletal elements (i.e. α-syntrophin) or basal membrane components (i.e. agrin) could be disturbed, resulting in the instability of the perivascular aggregation state of AQP4 as reported earlier [44] and probably together with other proteins to influence the ability of astrocytes to contribute to maintaining the integrity of the BBB [46].

An important question is related to the consequences of the reduction of AQP4 protein in relationships to peritumoral edema in GBM. Is the reduction and the concomitant relocation of AQP4 a negative effect that causes or increment the edema formation? Or is it a way to resolve the vasogenic edema that constantly occurs in GBM? Expression analysis show that AQP4 downregulation in T regions is not accompanied by a parallel reduction in Pt regions. Only levels of AQP4ex weighted to total AQP4 were found to be significantly affected. Importantly, this reduction is also complemented by an increase of the M1ex expression which, taking into account the demonstrated negative effect of M1 in OAP formation, should further destabilize the polarization of AQP4. IF data confirm a strong increase in AQP4 in parenchymal membranes, which are normally immuno-negative or weakly positive. Reduced vascular-related polarity of AQP4 implies the presence of water channels in physiologically unsuitable membrane domains. BBB breakdown with influx of water into the brain parenchyma will result in the incapability of the astrocytes to direct the release of water out of the interstitial space into the vascular space where AQP4 is no longer present. In this phenomenon AQP4ex assumes a strategic importance and proves the physiological importance of the readthrough isoform in the correct anchoring of AQP4, as well its involvement in GBM and in many other pathological situations in which AQP4 polarization is lost.

We can conclude that AQP4ex plays a key role in the human brain in the anchoring of the canonical isoforms to the perivascular pole. Therefore, the reduction in AQP4ex, leading to reduction and delocalization of AQP4, and to a subtle alteration of AQP4 membrane organization is likely to undermine the integrity of the BBB. Thus, the barrier losing its normal microenvironment conformation may be involved in the accumulation of edema in the peritumoral tissue. Finally, AQP4ex could be considered as a potential new early biomarker of GBM progression and a target for AQP4 modulation [14].

## Supplementary Information

Below is the link to the electronic supplementary material.

Supplementary file1 (DOCX 156 KB)

## Data Availability

All data generated or analyzed during this study are included in this published article.

## References

[1] Acerbi F, Broggi M, Eoli M, Anghileri E, Cavallo C, Boffano C, Cordella R, Cuppini L, Pollo B, Schiariti M (2014). Is fluorescein-guided technique able to help in resection of high-grade gliomas?. Neurosurg Focus.

[2] Amiry-Moghaddam M, Otsuka T, Hurn PD, Traystman RJ, Haug FM, Froehner SC, Adams ME, Neely JD, Agre P, Ottersen OP (2003). An alpha-syntrophin-dependent pool of AQP4 in astroglial end-feet confers bidirectional water flow between blood and brain. Proc Natl Acad Sci USA.

[3] Barbagallo G, Maione M, Peschillo S, Signorelli F, Visocchi M, Sortino G, Fiumanò G, Certo F (2019). Intraoperative computed tomography, navigated ultrasound, 5-amino-levulinic acid fluorescence and neuromonitoring in brain tumor surgery: overtreatment or useful tool combination?. J Neurosurg Sci.

[4] Becker AP, Sells BE, Haque SJ, Chakravarti A (2021). Tumor heterogeneity in glioblastomas: from light microscopy to molecular pathology. Cancers.

[5] De Bellis M, Pisani F, Mola MG, Basco D, Catalano F, Nicchia GP, Svelto M, Frigeri A (2014). A novel human aquaporin-4 splice variant exhibits a dominant-negative activity: a new mechanism to regulate water permeability. Mol Biol Cell.

[6] De Bellis M, Pisani F, Mola MG, Rosito S, Simone L, Buccoliero C, Trojano M, Nicchia GP, Svelto M, Frigeri A (2017). Translational readthrough generates new astrocyte AQP4 isoforms that modulate supramolecular clustering, glial endfeet localization, and water transport. Glia.

[7] Diaz RJ, Dios RR, Hattab EM, Burrell K, Rakopoulos P, Sabha N, Hawkins C, Zadeh G, Rutka JT, Cohen-Gadol AA (2015). Study of the biodistribution of fluorescein in glioma-infiltrated mouse brain and histopathological correlation of intraoperative findings in high-grade gliomas resected under fluorescein fluorescence guidance. J Neurosurg.

[8] Ding Y, Liu J, Xu Y, Dong X, Shao B (2020). Evolutionary adaptation of aquaporin-4 in yak (*Bos grunniens*) brain to high-altitude hypoxia of Qinghai-Tibetan Plateau. High Alt Med Biol.

[9] Emami Nejad A, Najafgholian S, Rostami A, Sistani A, Shojaeifar S, Esparvarinha M, Nedaeinia R, Haghjooy Javanmard S, Taherian M, Ahmadlou M (2021). The role of hypoxia in the tumor microenvironment and development of cancer stem cell: a novel approach to developing treatment. Cancer Cell Int.

[10] Frigeri A, Gropper MA, Turck CW, Verkman AS (1995). Immunolocalization of the mercurial-insensitive water channel and glycerol intrinsic protein in epithelial cell plasma membranes. Proc Natl Acad Sci USA.

[11] Frigeri A, Gropper MA, Umenishi F, Kawashima M, Brown D, Verkman AS (1995). Localization of MIWC and GLIP water channel homologs in neuromuscular, epithelial and glandular tissues. J Cell Sci.

[12] Frigeri A, Nicchia GP, Nico B, Quondamatteo F, Herken R, Roncali L, Svelto M (2001). Aquaporin-4 deficiency in skeletal muscle and brain of dystrophic mdx mice. FASEB J.

[13] Frigeri A, Nicchia GP, Repetto S, Bado M, Minetti C, Svelto M (2002). Altered aquaporin-4 expression in human muscular dystrophies: a common feature?. FASEB J.

[14] Frigeri A, Nicchia GP, Svelto M (2007). Aquaporins as targets for drug discovery. Curr Pharm Des.

[15] Hoddevik EH, Khan FH, Rahmani S, Ottersen OP, Boldt HB, Amiry-Moghaddam M (2017). Factors determining the density of AQP4 water channel molecules at the brain-blood interface. Brain Struct Funct.

[16] Isoardo G, Morra I, Chiarle G, Audrito V, Deaglio S, Melcarne A, Junemann C, Naddeo M, Cogoni M, Valentini MC (2012). Different aquaporin-4 expression in glioblastoma multiforme patients with and without seizures. Mol Med.

[17] Jin BJ, Rossi A, Verkman AS (2011). Model of aquaporin-4 supramolecular assembly in orthogonal arrays based on heterotetrameric association of M1–M23 isoforms. Biophys J.

[18] Lemée JM, Clavreul A, Menei P (2015). Intratumoral heterogeneity in glioblastoma: don't forget the peritumoral brain zone. Neuro Oncol.

[19] Liu S, Mao J, Wang T, Fu X (2017). Downregulation of aquaporin-4 protects brain against hypoxia ischemia via anti-inflammatory mechanism. Mol Neurobiol.

[20] Loughran G, Chou MY, Ivanov IP, Jungreis I, Kellis M, Kiran AM, Baranov PV, Atkins JF (2014). Evidence of efficient stop codon readthrough in four mammalian genes. Nucleic Acids Res.

[21] Marcu L, Jo JA, Butte PV, Yong WH, Pikul BK, Black KL, Thompson RC (2004). Fluorescence lifetime spectroscopy of glioblastoma multiforme. Photochem Photobiol.

[22] McCoy E, Sontheimer H (2007). Expression and function of water channels (aquaporins) in migrating malignant astrocytes. Glia.

[23] Mou K, Chen M, Mao Q, Wang P, Ni R, Xia X, Liu Y (2010). AQP-4 in peritumoral edematous tissue is correlated with the degree of glioma and with expression of VEGF and HIF-alpha. J Neurooncol.

[24] Nicchia GP, Cogotzi L, Rossi A, Basco D, Brancaccio A, Svelto M, Frigeri A (2008). Expression of multiple AQP4 pools in the plasma membrane and their association with the dystrophin complex. J Neurochem.

[25] Nicchia GP, Rossi A, Nudel U, Svelto M, Frigeri A (2008). Dystrophin-dependent and -independent AQP4 pools are expressed in the mouse brain. Glia.

[26] Nico B, Mangieri D, Tamma R, Longo V, Annese T, Crivellato E, Pollo B, Maderna E, Ribatti D, Salmaggi A (2009). Aquaporin-4 contributes to the resolution of peritumoural brain oedema in human glioblastoma multiforme after combined chemotherapy and radiotherapy. Eur J Cancer.

[27] Nielsen S, Nagelhus EA, Amiry-Moghaddam M, Bourque C, Agre P, Ottersen OP (1997). Specialized membrane domains for water transport in glial cells: high-resolution immunogold cytochemistry of aquaporin-4 in rat brain. J Neurosci.

[28] Noell S, Ritz R, Wolburg-Buchholz K, Wolburg H, Fallier-Becker P (2012). An allograft glioma model reveals the dependence of aquaporin-4 expression on the brain microenvironment. PLoS ONE.

[29] Noell S, Wolburg-Buchholz K, Mack AF, Ritz R, Tatagiba M, Beschorner R, Wolburg H, Fallier-Becker P (2012). Dynamics of expression patterns of AQP4, dystroglycan, agrin and matrix metalloproteinases in human glioblastoma. Cell Tissue Res.

[30] Palazzo C, Buccoliero C, Mola MG, Abbrescia P, Nicchia GP, Trojano M, Frigeri A (2019). AQP4ex is crucial for the anchoring of AQP4 at the astrocyte end-feet and for neuromyelitis optica antibody binding. Acta Neuropathol Commun.

[31] Pavlov V, Meyronet D, Meyer-Bisch V, Armoiry X, Pikul B, Dumot C, Beuriat PA, Signorelli F, Guyotat J (2016). Intraoperative probe-based confocal laser endomicroscopy in surgery and stereotactic biopsy of low-grade and high-grade gliomas: a feasibility study in humans. Neurosurgery.

[32] Picart T, Armoiry X, Berthiller J, Dumot C, Pelissou-Guyotat I, Signorelli F, Guyotat J (2017). Is fluorescence-guided surgery with 5-ala in eloquent areas for malignant gliomas a reasonable and useful technique?. Neurochirurgie.

[33] Pisani F, Cammalleri M, Dal Monte M, Locri F, Mola MG, Nicchia GP, Frigeri A, Bagnoli P, Svelto M (2018). Potential role of the methylation of VEGF gene promoter in response to hypoxia in oxygen-induced retinopathy: beneficial effect of the absence of AQP4. J Cell Mol Med.

[34] Pisani F, Rossi A, Nicchia GP, Svelto M, Frigeri A (2011). Translational regulation mechanisms of aquaporin-4 supramolecular organization in astrocytes. Glia.

[35] Rohlmann A, Gocht A, Wolburg H (1992). Reactive astrocytes in myelin-deficient rat optic nerve reveal an altered distribution of orthogonal arrays of particles (OAP). Glia.

[36] Rosito S, Nicchia GP, Palazzo C, Lia A, Buccoliero C, Pisani F, Svelto M, Trojano M, Frigeri A (2018). Supramolecular aggregation of aquaporin-4 is different in muscle and brain: correlation with tissue susceptibility in neuromyelitis optica. J Cell Mol Med.

[37] Saadoun S, Papadopoulos MC, Davies DC, Krishna S, Bell BA (2002). Aquaporin-4 expression is increased in oedematous human brain tumours. J Neurol Neurosurg Psychiatry.

[38] Semenza GL (2012). Hypoxia-inducible factors: mediators of cancer progression and targets for cancer therapy. Trends Pharmacol Sci.

[39] Simone L, Pisani F, Mola MG, De Bellis M, Merla G, Micale L, Frigeri A, Vescovi AL, Svelto M, Nicchia GP (2019). AQP4 aggregation state is a determinant for glioma cell fate. Cancer Res.

[40] Smith AJ, Jin BJ, Ratelade J, Verkman AS (2014). Aggregation state determines the localization and function of M1- and M23-aquaporin-4 in astrocytes. J Cell Biol.

[41] Stokum JA, Mehta RI, Ivanova S, Yu E, Gerzanich V, Simard JM (2015). Heterogeneity of aquaporin-4 localization and expression after focal cerebral ischemia underlies differences in white versus grey matter swelling. Acta Neuropathol Commun.

[42] Suero Molina EJ, Ardon H, Schroeteler J, Klingenhöfer M, Holling M, Wölfer J, Fischer B, Stummer W, Ewelt C (2013). Aquaporin-4 in glioma and metastatic tissues harboring 5-aminolevulinic acid-induced porphyrin fluorescence. Clin Neurol Neurosurg.

[43] Vitovcova B, Skarkova V, Rudolf K, Rudolf E (2020). Biology of glioblastoma multiforme-exploration of mitotic catastrophe as a potential treatment modality. Int J Mol Sci.

[44] Warth A, Kröger S, Wolburg H (2004). Redistribution of aquaporin-4 in human glioblastoma correlates with loss of agrin immunoreactivity from brain capillary basal laminae. Acta Neuropathol.

[45] Warth A, Simon P, Capper D, Goeppert B, Tabatabai G, Herzog H, Dietz K, Stubenvoll F, Ajaaj R, Becker R (2007). Expression pattern of the water channel aquaporin-4 in human gliomas is associated with blood-brain barrier disturbance but not with patient survival. J Neurosci Res.

[46] Wolburg H, Noell S, Fallier-Becker P, Mack AF, Wolburg-Buchholz K (2012). The disturbed blood-brain barrier in human glioblastoma. Mol Aspects Med.

[47] Yamamoto N, Yoneda K, Asai K, Sobue K, Tada T, Fujita Y, Katsuya H, Fujita M, Aihara N, Mase M (2001). Alterations in the expression of the AQP family in cultured rat astrocytes during hypoxia and reoxygenation. Brain Res Mol Brain Res.

[48] Zhao WJ, Zhang W, Li GL, Cui Y, Shi ZF, Yuan F (2012). Differential expression of MMP-9 and AQP4 in human glioma samples. Folia Neuropathol.

