# Supplementary material for: Alteration of the translational readthrough isoform AQP4ex induces redistribution and downregulation of AQP4 in human glioblastoma
Source: Cell Mol Life Sci. 2022 Feb 20;79(3):140. doi: 10.1007/s00018-021-04123-y (PMC8858924; doi:10.1007/s00018-021-04123-y)
Supplement: Supplementary file 1 — Supplementary file1 (DOCX 156 KB) [file 18_2021_4123_MOESM1_ESM.docx]

**SUPPLEMENTARY DATA**

**Figure S1**: Evaluation of the expression of AQP4 and AQP4ex indifferent brain regions. AQP4 expression levels (top) and AQP4ex (M23ex, bottom), relative to the global AQP4 expression in GBM biopsies grouped in three different areas (Frontal, Temporal and extensive). (*p<0.05,**p<0.001: Kruskal-Wallis, Post test: Dunn's multiple comparisons test).


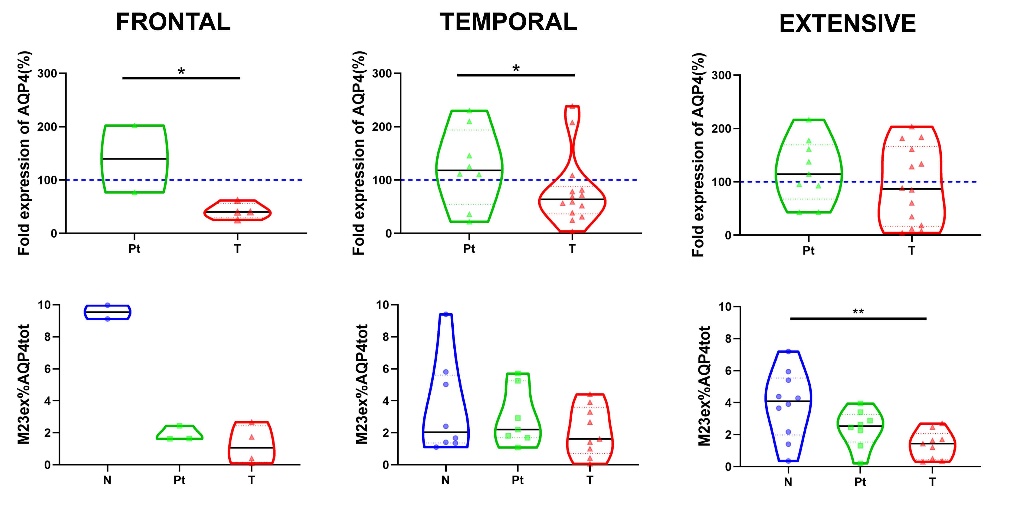


**Figure S2**: Fluorescein analysis of control of treated GBM samples (SF-GBM), compared with untreated controls (Non treated).Scatter plot, report the median of the concentration (pg/ml) of the Sodium Fluoescein (SF) in each samples. Note an higher concentration in treated GBM samples (*blue*) compared to No-treated controls (*red*). (*p<0.05; Kruskal-Wallis; Dunn’mutliple comparisons test).

**
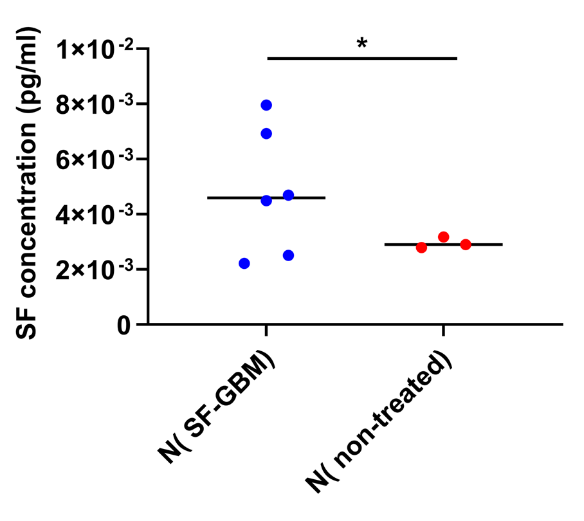
**
